# Prevalence of *fimA* genotypes of *Porphyromonas gingivalis* in adolescent orthodontic patients

**DOI:** 10.1371/journal.pone.0188420

**Published:** 2017-11-27

**Authors:** Shuang Pan, Yi Liu, Yi Si, Qiang Zhang, Lin Wang, Jianwei Liu, Chunling Wang, Shuiqing Xiao

**Affiliations:** 1 Department of Orthodontics, School of Stomatology, Shandong University, Jinan, China; 2 Department of Orthodontics, Jinan Stomatological Hospital, Jinan, China; 3 Pediatric Research Institute, Qilu Children’s Hospital of Shandong University, Jinan, China; 4 Department of Oral Medicine, Shandong Medical College, Jinan, China; 5 Department of Implantology, Jinan Stomatological Hospital, Jinan, China; 6 Department of Stomatology, the First Hospital of Jinan, Jinan, China; University of the Pacific, UNITED STATES

## Abstract

**Background:**

The placement of fixed orthodontic appliances may alter the composition of oral microbiota and has the potential risk of periodontal complication. *Porphyromonas gingivalis* fimbriae play a critical role in colonization of *P*. *gingivalis* in subgingival regions. In this study, we investigated the association between the prevalence of *P*. *gingivalis*-specific *fimA* genotypes and periodontal health status in adolescent orthodontic patients, to identify the pathogencity of *P*. *gingivalis* during orthodontic therapy.

**Methods:**

Sixty-one adolescent orthodontic patients were enrolled in the case group, while the control group consisted of 56 periodontally healthy adolescents. At baseline (T0), clinical parameter (gingival index) was tested, and subgingival plaque samples were obtained from the lower incisors. The incidences of *P*. *gingivalis* and *fimA* genotypes were detected by polymerase chain reaction. All parameters were reassessed after 1 month (T1), 2 months (T2), 3 months (T3), and 6 months (T4) in the case group and then compared with those of the controls.

**Results:**

Both microbiological and clinical parameters from orthodontic patients started to increase after placement of fixed appliances. Maximum values were reached at 3 months after placement and followed by their decreases at six months. However, the microbiological and clinical parameters in the case group were significantly higher than those of the control group. The GI of *fimA* II, IV-positive samples was significantly higher than that of negative samples.

**Conclusion:**

*P*. *gingivalis* carrying *fimA* II or IV was closely related to orthodontic gingivitis. In addition, proper oral hygiene control could lead to little increase in dental plaque accumulation, and exert a beneficial effect to periodontal tissues.

## Introduction

Fixed orthodontic therapy is the most effective method in contemporary orthodontics, however, it may cause periodontal complication due to hampering oral hygiene and altering the nature of dental plaque [1]. The overall incidence of gingivitis associated with orthodontic therapy was 56.8% in adolescents and 34.4%in adult in China, respectively [2].

It has been confirmed that anaerobic microbiota in subgingival plaque are the primary agents in the initiation and progression of periodontal diseases [1]. Previous studies by our team and other researchers demonstrated that there were positive associations between periodontal diseases and several pathogens, including *A*. *actinomycetemcomitans*, *P*. *gingivalis*, *P*. *intermedia*, and *T*. *forsythia* [1, 3–6]. *P*. *gingivalis* is considered as a putative etiological agent in different types of periodontal diseases, whose virulence attributes to its various surface components, such as lipopolysaccharide, capsule, gingipain, fimbria and so on [6–9]. Among them, the major fimbrial subunit, fimbrillin, is thought to play critical roles in the colonization of *P*. *gingivalis* in periodontal tissues, as it promotes bacterial adhesion to and invasion to subgingival regions [10–15]. The *P*. *gingivalis fimA* gene, which encodes fimbrillin, is a single copy on the bacterial chromosome and no homologous sequences were detected in other gram-negative oral anaerobe [16]. *FimA* has been classified into six variants (types I–V, Ib) according to clonal variations of its nucleotide sequences [17–19]. Accumulating evidences suggested that *P*. *gingivalis* strains with different *fimA* genotypes displayed distinct virulence features [20, 21]. According to the diversity of pathogenicity, there are two groups in *P*. *gingivalis* stains, disease related and non-disease related, and the variance of genetic heterogeneity was the base of the differential of pathogenicity in microorganisms [22, 23]. Analysis of the different *fimA* genotypes and their clonal distribution may provide insight into the epidemiology of periodontal diseases.

It is demonstrated that fixed appliances could alter the equilibrium of the microorganism ecosystem, and increase the colonization of pathogenic bacteria which is responsible for periodontal diseases [1, 24, 25]. Our previous research showed that the prevalence of *P*. *gingivalis* detected at two months after orthodontic therapy was as much as 31.11%higher than baseline and the count of *P*. *gingivalis* assessed immediately after removing orthodontic appliances was obviously higher than that of healthy group [1, 26]. However, there was no report on the variation in distribution of *fimA* genotypes during the orthodontic therapy.

In this case, we assessed the prevalence of *P*. *gingivalis* and *fimA* genotypes in subgingival plaque samples among different time points after placing orthodontic appliances, to identify the pathogencity of *P*. *gingivalis* with different *fimA* genotypes during orthodontic therapy.

## Materials and methods

### Ethics statement

The human subject protocol was approved by the Ethics Committee of Jinan Stomatological Hospital and School of Stomatology, Shandong University, and written informed consents were provided by the patients of each participant.

### Subjects

Subjects of both case and control groups were consecutively recruited from Department of Orthodontics, Jinan Stomatological Hospital during June 2015- October 2015 with same procedure and criteria as described previously [1]. In short, 107periodontally healthy adolescents who referred to our hospital for orthodontic treatment were enrolled in this study and randomly assigned to be the case group and control group. Among them, sixty-one subjects (22 males and 39 females, mean age 13.80±1.61 years, ranging from 11 to 17 years, S1 Table) were classified into the case group and 56 subjects (16 males and 40 females, mean age 14.24±1.30 years, ranging from 12 to 17 years) were grouped as the controls. Both groups matched for age and sex. All participants met the following criteria: (1) a healthy gingival condition defined as a periodontal probing depth of less than 4mm and a gingival index less than 1, (2) no smoking, (3) no known systemic disease, (4) no alveolar bone loss visible on X-ray, (5) absence of fixed restorations or removal partial dentures, (6) no use of antibiotics within 3 months before the study, (7) no periodontal therapy within the previous 6 months. [1].

All subjects in the case group were treated with fixed buccal metal appliances (Victory, 3M Unitek, Calif) on incisors, premolars, and molars. The arch wires were ligated with elastomeric ligature. The brackets were placed on both jaws when the first clinical periodontal examination was done. The oral hygiene instructions (tooth-bushing Bass skill) and correct method of using tooth brush were provided for all case subjects before placing brackets (S1 Appendix).

### Bacteria strains

The reference strains of *P*. *gingivalis* (ATCC33277), *F*. *nucleatum* (ATCC25586) and *A*. *actinomycetemcomitans* (ATCC29522) were obtained from The State Key Laboratory of Oral Diseases, Sichuan University (Chengdu, China).

### Clinical examination and gingival index

Before sample collection, the dentist evaluated Gingival index (GI) of both groups at the teeth of 31, 32, 41 and 42 with same procedure as described previously [1]. The value of GI for each subject was the mean measurement of the four teeth. In the case group, measurement were made at five time points: Baseline T_0_ = before appliances placement, T_1_ = one month after bonding, T_2_ = two months after bonding, T_3_ = three months after bonding, and T_4_ = six months after bonding; while in the control group, the GI values were only measured at baseline (T0) and compared with those of the case group.

Periodontal examination and sample collection were carried out by the same dentist.

### Microbial sampling and DNA extraction

Sampling procedure was described before [1]. In brief, subgingival microbial samples were collected at the same sites after measuring the GI. Samples were obtained from 10:00 to 12:00 a.m. and at least two hours after eating, drinking, or taking oral hygiene measures, and then careful removal of supragingival plaque and saliva, the sites were isolated with sterile cotton rolls, and gently air dried. A sterile filter paper strip (ISO 30) was softly inserted into the bottom of periodontal pockets for 30 seconds until a minimum of resistance. Four paper points from each subject were immediately placed into a sterile microcentrifuge tube containing 0.5 ml of PBS [1, 27, 28]. The tubes were stored at -20°C until analyzed. The bacterial DNA was extracted by the boiling method [29]. Briefly, a 10 μl aliquot of each stored sample was added to 10μl of 2 ×lysis buffer (2 mM EDTA, 1%X-100). The mixture was boiled for 10 minutes and then placed on ice. The supernatant was used as the template for PCR amplification.

### Specificity of the 16S rRNA-based PCR

Specificity of the 16S rRNA-based PCR was evaluated by using specific primers of 16SrRNA gene and the reference strains, including *P*. *gingivalis* (ATCC33277), *F*. *nucleatum* (ATCC25586), and *A*. *actinomycetemcomitans* (ATCC29522). The amplified products from clinical samples were randomly chosen for sequencing.

### The 16S rRNA-based PCR and *fimA* genotypes specific PCR

The 16S rRNA-based PCR was used to determine the prevalence of *P*. *gingivalis in* subgingival biofilm. The specific primers of *fimA* genotypes designed previously [18, 30] were used for *fimA* typing and detection from positive samples of *P*. *gingivalis* (Table 1). PCR amplification was carried out in a Tetrad Thermal Cycler (MJ Research, South San Francisco, USA) according to our previous description [6, 28] and run in a 25μlreaction mixture, including 4.5μl 10×PCR buffer (100 mM Tris-HCl, 500 mM KCl, and 15 mM MgCl_2_), 0.25 mM of each deoxynucleoside triphosphate (dNTP), 10μM of each primers, 5μl of template DNA, and 1.5 units of Taq DNA polymerase (Tiangen Biotech, Beijing), and sterile distilled water. Each sample was amplified for 5 min at 94°Cand 30 cycles, with each cycle consisting of denaturation at 94°C for 30 sec, annealing at 58°C for 30 sec, extension at 72°C for 1 min, and final extension for 10 min. The amplified products were then electrophoresed on 1.5% agarose gel in Tris-acetate buffer (40 mM Tris acetate, 1 mM EDTA, pH8.0). The products were visualized with ethidium bromide by UV transillumination. (dx.doi.org/10.17504/protocols.io.jskcncw)

**Table 1:** The primers of *P*. *gingivalis* and *fimA*.

Primers	Sequence(5'-3')	Annealing temp(°C)	Sizes(bp)
Pg F	AGGCAGCTTGCCATACTGCG	57	404
Pg R	ACTGTTAGCAACTACCGATGT		
FimA I F	CTGTGTGTTTATGGCAAACTTC	58	392
FimA I R	AACCCCGCTCCCTGTATTCCGA		
FimA Ib F	CAGCAGAGCCCAAAAACAATCG	54	271
FimA Ib R	TGTCAGATAATTAGCGTCTGC		
FimA II F	ACAACTATACTTATGACAATGG	56	257
FimA II R	AACCCCGCTCCCTGTATTCCGA		
FimA III F	ATTACACCTACACAGGTGAGGC	58	247
FimA III R	AACCCCGCTCCCTATATTCCGA		
FimA IV F	CTATTCAGGTGCTATTACCCAA	58	251
FimA IV R	AACCCCGCTCCCTGTATTCCGA		
FimA V F	AACAACAGTCTCCTTGACAGTG	50	462
FimA V R	TATTGGGGGTCGAACGTTACTGTC		

The products of six *fimA* genotypes were randomly sequenced on 3730 sequencer (Applied Biosystems, Foster City, CA, USA) to confirm the validity of the specific *fimA* PCR in *P*. *gingivalis* positive samples. The alignment of the resulting sequences was carried out with BLAST (http://www.ncbi.nlm.nih.gov/BLAST)

The primers for amplification of *16S rRNA* gene and *fimA* gene were listed in Table 1.

### Statistical analysis

The data were analyzed by using the SPSS statistical software (version 20.0, SPSS, Chicago, IL). The Mann-Whitney U test and the Wilcoxon signed rank test were used to detect significant intergroup and intragroup differences regarding GI. Chi-squared test was used to compare detection rates of *P*. *gingivalis* and *fimA* genotypes between two groups. The McNemar test was utilized to compare the prevalence of *P*. *gingivalis* and *fimA* genotypes in case group. The Mann-Whitney U test was used to determine the correlation between prevalence of *fimA* genotypes and GI in orthodontic patients. The significance for all statistical tests was predetermined at *P*<0.05.

## Results

### Detection and confirmation of 16S rRNA-based PCR for *P*. *gingivalis*

The reference stains were first amplified by the16S rRNA-based PCR to evaluate its specificity. The positive product appeared only from *P*. *gingivalis* (ATCC33277), not from *F*. *nucleatum* (ATCC25586) and *A*. *actinomycetemcomitans* (ATCC29522).

At baseline (T_0_), the frequency of *P*. *gingivalis* in from 117 subgingival samples of the two groups was twenty-nine (24.79%), of which thirteen (23.21%) from the control group and sixteen (26.23%) from the case group. From T_0_ to T_3_, the detection rate of *P*. *gingivalis* increased significantly in the case group: 25 (42.62%) at T_1_, 31 (50.82%) at T_2_ and 51 (83.61%) at T_3_. At T_4_, the detection of *P*. *gingivalis* decreased to 26 (42.62%). The detection rate of *P*. *gingivalis* at different time points after wearing appliances was significantly higher than that of T_0_ in the case group and control group: T_1_ (P<0.05), T2 (P<0.01), T_3_ (P<0.01) and T4 (P<0.05) (Table 2).

**Table 2:** The prevalence of *P*. *gingivalis* and *fimA* genotypes in two groups.

	control group	case group				
	T_0_	T_0_	T_1_	T_2_	T_3_	T_4_
*P*. *gingivalis*	13 (23.21)	16 (26.23)	25 (42.62)*^†^	31 (50.82)**^††^	51 (83.61)**^††^	26 (42.62)*^†^
I	6 (10.71)	7 (11.48)	7 (11.48)	7 (11.48)	11 (18.03)	9 (14.75)
Ib	3 (5.36)	3 (4.92)	4 (6.56)	3 (4.92)	5 (8.20)	2 (3.28)
II	2 (3.57)	2 (3.28)	4 (6.56)	5 (8.20)	9 (14.75)*^†^	8 (13.11)
III	4 (7.14)	2 (3.28)	3 (4.92)	4 (6.56)	7 (11.48)	7 (11.48)
IV	2 (3.57)	2 (3.28)	3 (4.92)	7 (11.48)	15 (24.59)**^††^	10 (16.39)*^†^
V	5 (8.93)	6 (9.84)	6 (9.84)	14 (22.95)	22 (36.07)**^†^	13 (21.31)
untypeable	3 (5.36)	5 (8.20)	5 (8.20)	8 (13.11)	8 (13.11)	4 (6.56)

Chi-squared test was used to compare detection rates of *P*. *gingivalis* and *fimA* genotypes between two groups.

* *P*<0.05,

** *P*<0.01.

The McNamara test was utilized to compare the prevalence of *P*. *gingivalis* and *fimA* genotypes in case group*s*.

^†^ *P*<0.05,

^††^ *P*<0.01.

Ten *P*. *gingivalis* positive samples were randomly selected for sequencing in Invitrogen Company (Invitrogen, Shanghai) to confirm the validity of the 16S rRNA-based PCR in clinical subgingival biofilm samples.

### Detection and confirmation of 16S rRNA-based PCR for *fimA* genotypes

The distribution of *fimA* genotypes among *P*. *gingivalis*-positive samples was subsequently analyzed (Table 2). The distribution of *fimA* genotypes in the case group showed no significant change over time from T_0_ to T_2_, and there was no difference between two groups (*P*>0.05). At T_3_, the detection rate was 14.75% for *fimA* type II (*P*<0.05), 24.5% for *fimA* type IV (*P*<0.01), and 36.07% for *fimA* type V (*P*<0.01), which was found at higher level than that of the controls (3.57% for *fimA* type II and IV, 8.93% for type V) and T_0_ in the case group (3.28% for *fimA* type II and IV, 9.84% for type V). At T_4_, the detection rate of *fimA* type II and V decreased to 13.11% and 21.31%, and there was no significant difference in the detection of *fimA* type I, II, III and V between two groups. Only *fimA* type IV (16.39%) was detected higher than that of T_0_ in both groups (*P*<0.05).

### Association of *fimA* genotypes and GI

The Mann-Whitney U test was used to determine the correlation between prevalence of *fimA* genotypes and GI in case group. As *P*. *gingivalis*-positive and *fimA*-positive samples at T_3_ were the largest than those of other time points. We used the data of T_3_ to assess the correlation of *fimA* genotypes and GI (shown in Table 3), regarding GI, there was no significant difference between *fimA* I/ III/ V/ Ib-positive samples and negative samples (*P*>0.05). However, the GI of *fimA* II, IV-positive samples was significantly higher than that of negative samples (*P*<0.01).

**Table 3:** The correlation of *fimA* genotypes and GI.

		GI (n %)			*P*
		0	1	2	
*fimA* I	negative	14 (28.0)	28 (56.0)	8 (16.0)	0.13
	positive	5 (45.5)	6 (54.5)	0 (0.0)	
*fimA* II	negative	19 (36.5)	30 (57.7)	3 (5.8)	<0.001
	positive	0 (0.0)	4 (44.4)	5 (55.6)	
*fimA* III	negative	17 (31.5)	29 (53.7)	8 (14.8)	0.713
	positive	2 (28.6)	5 (71.4)	0 (0.0)	
*fimA* IV	negative	19 (41.3)	22 (47.8)	5 (10.9)	0.006
	positive	0 (0.0)	12 (80.0)	3 (20.0)	
*fimA* V	negative	13 (33.3)	22 (56.4)	4 (10.3)	0.438
	positive	6 (27.3)	12 (54.5)	4 (18.2)	
*fimA* Ib	negative	18(32.1%)	31(55.4%)	7(12.5%)	0.239
	positive	1(20%)	3(50%)	1(20%)	

The Mann-Whitney U test was used to determine the correlation between prevalence of *fimA* genotypes and GI in case group.

### Evaluation of clinical parameter

It was showed that the value of GI (Table 4) in case group increased after placing appliances and reached to the maximum at T_3_, then decreased at T_4_. The GI at T_4_ was found higher than that of baseline in two groups (*P*<0.01), but obviously lower than that at T_3_(*P*<0.01).

**Table 4:** GI from the orthodontic patients and peridontally healthy subjects.

	control group	case group				
	T_0_	T_0_	T_1_	T_2_	T_3_	T_4_
GI 0	56	61	36	29	19	24
GI 1	0	0	25	30	34	25
GI 2	0	0	0	2	8	2

## Discussion

Saxe et al. and Lindhe et al. indicated that gingivitis might represent a pre-phase in adults [31, 32]. Morinushi et al [33] investigated the association between gingivitis and colonization of *P*.*gingivalis* in children of different ages demonstrated that the children carrying detectable levels of *P*. *gingivalis* had significantly higher inflammation scores than those with negative *P*. *gingivalis*. Furthermore, they demonstrated that *P*. *gingivalis* was related to the progression of gingivitis and the onset of the periodontitis in adolescents (12 years and older). There were few documents examining the colonization by *P*. *gingivalis* in a long term utilization of fixed orthodontic appliances (extending to six months) in adolescents. A previous research showed various periodontal pathogens increased significantly after 6 months of fixed appliance treatment, but returned to pretreatment levels by 12 months of orthodontic treatment [25]. Ristic et al. found the detection rate of *P*. *gingivalis* increased from baseline to three months after bonding, then decreased at six months, which varied with the change of GI [34]. In this case, not only the incidence of *P*. *gingivalis*, but also *fimA* II, IV and V continuously increased after appliances placement, and reached the maximum at T_3_, and then followed by a decrease at T_4_, which was consistent with the change of GI. This could be explained by improvement of oral hygiene and reestablishment of host-microorganisms balance. It was demonstrated that periodontal diseases resulted from a rupture of the dynamic balance between the pathogens and host defense system [1, 34]. Scientific investigations indicated that the presence of fixed orthodontic appliances would hamper effective oral hygiene and result in an ecological environment favorable to accumulate gram-negative anaerobic bacteria in subgingival regions, especially for adolescents [34, 35]. For this reason, the host–microorganisms balance is broken, which leads to periodontal diseases. Guo [35] investigated the differences of the impact of orthodontic appliances on periodontal tissues between adults and children and found that the frequency of *P*. *gingivalis* presented stable trends among adults during the first three months, but increased significantly among children in the first three months. This article illustrated that the effect of fixed orthodontic appliance on periodontal tissues was more apparent in children than in adults. It seems that 3 months following placing orthodontic appliances, most adolescent patients raised awareness of oral hygiene and mastered tooth-brushing skills. Accordingly, the rebalance of host and microorganisms started to establish. This resulted in a reduction of periodontal pathogens, which was consistent with better periodontal status. It should also be addressed that proper hygiene controls during the therapy contributed to little increase in plaque accumulation and the rebalance of host-microorganisms, consequently [34].

It was reported that *P*. *gingivalis* was detected not only at a high frequency in patients with periodontitis, but also at a low frequency in periodontally healthy individuals without obvious gingival inflammation [36, 37]. Therefore, specific virulent genotypes of the pathogens might be a cause of periodontal diseases [20]. Accumulating evidences suggested that *P*. *gingivalis* harboring Type II and IV (Pg-II, IV) fimbriae were disease-related isolates, while Pg-I and III were non-disease-related. Some researchers evaluated *fimA* genotypes in animal trials, demonstrating that *fimA* genotype II, Ib, and IV could cause stronger infections and inflammatory changes compared to strains with *fimA* genotype I and III [22, 38, 39]. Results of several clinical researches also believed that nucleotide genetic variation was likely related to virulence. In chronic periodontitis, *P*. *gingivalis* strains harboring *fimA* genotype II, IV, and Ib were obviously more common than isolates with other genotypes [18, 20, 22, 30, 40–42]. Also, *P*. *gingivalis* carrying type II fimbriae was found to be the predominant *fimA* genotype in patients with periodontitis in several epidemiological surveys, while *fimA* genotype IV was more prevalent in the gingivitis group [8, 42]. Additionally, the previous views that genotype II, IV, and Ib were related to virulence were supported by *fimA* genotyping of cultured clinical isolates of *P*. *gingivalis* sampled from patients with chronic periodontitis [20, 41]. On the contrary, strains with *fimA* genotype I were the most prevalent among *P*. *gingivalis* positive in periodontally healthy subjects, followed by genotype V [20, 30].

We detected the prevalence of *fimA* genotypes during the orthodontic therapy. At T_3_, consistent with the maximum GI, there was a statistically higher incidence of *fimA* II, IV and V than that at T_0_ and periodontally healthy individuals, demonstrating that they present stronger virulence of *P*. *gingivalis*. Subsequently, *fimA* II and V returned to be normal at T_4_, while the incidence of *fimA* IV was significantly higher than that at T_0_ and periodontally healthy individuals, meanwhile the periodontal clinical parameter was improved, implying the virulence of *P*. *gingivalis* tended to be weakened. We inferred that this might also due to reestablishment of host–microorganisms balance and improvement of oral hygiene.

According to previous reports, *fimA* V was detected in 0~29.7% of healthy subjects and 0~17.4% of periodontitis patients. In this study, we found *fimA* V appeared in 8.93% of healthy subjects, similar to data obtained from other groups, and in 36.07% of patients at T_3_, which was higher than data from previous reports. Moreover, the incidence of *fimA* V at T_3_ was statistically higher than that at T_0_ and healthy subjects. In order to further explore whether *fimA* V was associated with orthodontic gingivitis, we analyzed the correlation of GI and incidence of *fimA* V. The data showed that the GI levels of *fimA* II or IV positive groups were significantly higher than that of negative groups, while there was no difference in the GI levels between *fimA* V positive and negative groups, demonstrating that only *fimA* II and IV were associated with orthodontic gingivitis. However, it is unclear why *Pg-fimA* V significant increase during fixed orthodontic therapy. Longitudinal studies with larger sample size of orthodontic patients are needed to disclose it.

## Conclusion

The incidence of *P*. *gingivalis*, *fimA* II, IV and V increased after appliances placement and reached the maximum at the first three months, and followed by a decrease. *P*. *gingivalis* carrying *fimA* II or IV was associated with gingivitis during orthodontic therapy. In addition, appropriate oral hygiene could lead to decrease accumulation of dental plaque, and exert a beneficial effect on periodontal tissues.

## Supporting information

S1 Table Demographic characteristics of subjects in this study. (DOCX)

S1 Appendix The oral hygiene protocol. (DOCX)
