# Supplementary material for: Prevalence of fimA genotypes of Porphyromonas gingivalis in adolescent orthodontic patients
Source: PLoS One. 2017 Nov 27;12(11):e0188420. doi: 10.1371/journal.pone.0188420 (PMC5703466; doi:10.1371/journal.pone.0188420)
Supplement: S1 Appendix — (DOCX) [file pone.0188420.s002.docx]

**S1 Appendix. The oral hygiene protocol**

The subjects were instructed to brush their teeth after meal and before sleep in Bass technique at least three times a day, three minutes each time. Pay more attention to cleaning the tooth cervix, lingual side and brackets. Dental floss and interdental brush were also recommended to keep oral hygiene. The identical oral hygiene instructions were given to patients during therapy. Professional scaling and polishing were not performed in case eradicating periodontal pathogens which could change the composition of dental plaque and results of the study.
