# Supplementary material for: Prevalence of fimA genotypes of Porphyromonas gingivalis in adolescent orthodontic patients
Source: PLoS One. 2017 Nov 27;12(11):e0188420. doi: 10.1371/journal.pone.0188420 (PMC5703466; doi:10.1371/journal.pone.0188420)
Supplement: S1 Table — (DOCX) [file pone.0188420.s001.docx]

**S1 Table. Demographic characteristics of subjects in this study.**

|  | case group | Control group | P |
| --- | --- | --- | --- |
| subjects(n) | 61 | 56 |  |
| mean age(y)±SD | 13.80±1.61 | 14.24±1.30 | 0.10 |
| Male | 22(39.3%) | 16(28.6%) | 1.00 |

Manuscript information: PONE-D-17-20342

========================

%MS_NUMBER%

%ARTICLE_TITLE% Prevalence of fimA genotypes of Porphyromonas gingivalis in adolescent orthodontic patients

%JOURNALFULLTITLE%	plos one

========================

Dear editor：

Thank you for giving us required Modifications for PONE-D-17-20342R2 (Prevalence of fimA genotypes of Porphyromonas gingivalis in adolescent orthodontic patients), which was accepted by PLOS ONE . I have made all the required changes according to journal requirements and uploaded three files, including tracked-changes version of manuscript, S1 Table, and S1 Appendix. However, maybe I have made a mistake in the process of task submission, and I'm not sure whether the three files (tracked-changes version of your manuscript, S1 Table, and S1 Appendix) have been uploaded successfully. I would be greatly appreciate if you could spend some of your time check the status of task completion for me, and give me a chance to upload revised manuscript if the submission was not successful.

With kind regards,

Shuang Pan
